# Pet Ownership, Depression, Anxiety, Well-Being, and Cognitive Functioning in Older Adults: Cross-Sectional Study

**DOI:** 10.2196/76289

**Published:** 2026-02-02

**Authors:** Nathália Saraiva de Albuquerque, Isabela Schmitz Klain, Luisa Sigaran Machado, Wagner de Lara Machado, Carmen Moret-Tatay, Tatiana Quarti Irigaray

**Affiliations:** 1 Faculty of Psychology, Pontifícia Universidade Católica do Rio Grande do Sul, Porto Alegre, Brazil; 2 Doctoral School, Catholic University of Valencia San Vicente Mártir, San Agustín 3, Esc. A, Entresuelo 1, Valencia, 46002, Spain, +3496 363 74 12

**Keywords:** depression, older adults, human-animal interaction, pets, successful aging

## Abstract

**Background:**

Depression, anxiety, and cognitive decline are prevalent concerns among older adults and can negatively affect their well-being. Pet ownership has been proposed as a potential protective factor, but inconsistencies remain in the current literature.

**Objective:**

The study aims to compare symptoms of depression, anxiety, and levels of psychological well-being between older pet owners and non–pet owners. Additionally, we compared the cognitive functioning and cognitive reserve between these 2 groups of older individuals.

**Methods:**

This cross-sectional study included 215 community-dwelling older adults aged ≥59 years (mean 69.13, SD 6.89). Participants completed a sociodemographic questionnaire, the Modified Telephone Interview for Cognitive Status, Cognitive Reserve Index Questionnaire, Mental Health Continuum-Short Form, Geriatric Depression Scale-15, and Geriatric Anxiety Inventory. The data were collected via video calls through WhatsApp and analyzed using the JASP software. Group comparisons (pet owners vs nonowners) were conducted using the Mann-Whitney *U* test, followed by rank-biserial correlation for effect sizes (α=.05).

**Results:**

The sample consisted of 114 (53.0%) older adults pet owners and 101 (46.9%) older adults who did not own pets. Among the pet owners, 77.2% (n=88) were female, and 57.9% (n=66) were married or in common-law marriages. In the non–pet owners’ group, 71.3% (n=72) were female, and 46.5% (n=47) were married or in common-law marriages. Pet owners showed lower depression symptoms (Geriatric Depression Scale-15: mean 2.33 vs 3.00 non-owners; *U*=4714, *P*=.02; *r_b_*=−0.18, 95% CI –0.326 to –0.028), indicating a small effect. No significant group differences were observed for anxiety (Geriatric Anxiety Inventory: mean 5.33 vs 4.92, *P*=.52), psychological well-being (Mental Health Continuum-Short Form: mean 68.21 vs 67.46, *P*=.43), cognitive performance (Modified Telephone Interview for Cognitive Status: mean 24.74 vs 24.30, *P*=.60), or cognitive reserve (Cognitive Reserve Index Questionnaire: mean 123.82 vs 123.28, *P*=.95).

**Conclusions:**

The pet owner group presents fewer symptoms of depression in comparison to the non–pet owner group. Although no differences were observed in anxiety, well-being, cognitive functioning, or cognitive reserve, these findings suggest that pet companionship correlates with better emotional outcomes in later life. Future longitudinal studies are needed to clarify causal pathways and examine whether the frequency and quality of interactions with pets influence these outcomes.

## Introduction

The global population is aging at an increasing rate. According to United Nations projections, the population aged 65 years and older is expected to exceed 2.2 billion by the end of the 2070s, and the number of people aged 80 years or older will surpass the population of infants by the mid-2030s [1]. The Brazilian population is following this trend. According to a survey by the Brazilian Institute of Geography and Statistics, life expectancy in Brazil has risen from 71.1 years in 2000 to 76.4 years in 2023 and is projected to reach 83.9 years by 2070. Over the same period, the proportion of older adults aged 60 years or more nearly doubled, increasing from 8.7% (15.2 million individuals) to 15.6% (33.0 million individuals), and approximately 37.8% of Brazil’s population is expected to be aged 60 years or older by 2070 [2].

The increase in life expectancy is partly due to improvements in health conditions [2]. However, living longer does not always mean living better, as the aging process is associated with a decline in the individual’s physical and mental capacity [3]. Furthermore, this process brings about a series of changes that can negatively impact the psychological health of older adults [4].

The mental health status of older adults presents considerable challenges. Recent evidence indicates that depression and anxiety are highly prevalent mental health conditions among older adults worldwide [5]. In Brazil, a national survey found that 13.2% of the community-dwelling individuals aged 60 years or older reported depressive symptoms [6]. Depressive symptoms in older adults are strongly associated with declines in physical health and quality of life [7,8]. Moreover, late-life depression poses a substantial public health challenge, increasing health care demands and adding to the social and economic burden of aging populations [9].

Regarding anxiety, in a sample of older adults from a city in southern Brazil, the prevalence of anxiety disorders reached 21.9% [10]. Anxiety in older adults is often underrecognized, despite its high global prevalence and its associations with comorbidity and cognitive decline [5,11].

Cognitive decline is another major challenge associated with aging and has become a growing global public health concern. Recent estimates indicate that approximately 55 million people worldwide are currently living with dementia, and this number is projected to increase to 139 million by 2050 [12]. In Brazil, a recent population−based study reported a prevalence of 5.8% for dementia and 8.1% for cognitive impairment, with projections suggesting that these figures may increase 5-fold over the next 3 decades [13].

However, not all individuals experience cognitive decline in the same way. Despite similar levels of brain pathology, some older adults maintain relatively preserved cognitive functioning, suggesting that certain factors may protect against or delay the onset of decline. One concept that helps explain these interindividual differences is cognitive reserve, which can be understood as a set of cognitive resources that an individual acquires throughout life, which can help delay declines caused by the process of healthy aging, brain damage from dementia, and slow the progression of neurodegenerative diseases [14]. Cognitive reserve has 2 theoretical models, passive and active. The passive model refers to the brain’s structural characteristics, providing resilience against healthy brain aging and pathologies [15]. The active model, on the other hand, relates to external factors—activities performed throughout life that provide cognitive stimulation and lead to the strengthening or creation of new neural networks [16].

The high prevalence of depression, anxiety, and cognitive decline in later life underscores the need to identify protective factors that promote psychological and cognitive health among older adults, and the companionship of an animal could serve as a resource to achieve this goal [4]. Pets are becoming increasingly valued in society [17], and although pet ownership tends to decline as people age [18], it is possible that with the aging population, there will be more older individuals owning a companion animal [19]. According to Meier and Maurer [18], promoting pet ownership may contribute to enhancing individual well-being in older adults. Companion animals also appear to alleviate psychological symptoms in their owners, as pet ownership has been associated with fewer depressive symptoms, and regular interaction with a dog has been related to lower levels of anxiety [20,21]. Additionally, evidence suggests that pet ownership contributes to cognitive functioning in older adults and may help slow the rate of decline in executive functioning and episodic memory [22,23].

However, other studies do not support these findings. For example, the research by Hansen et al [24] found no relationship between pet ownership and psychosocial factors in adults aged 40 years and over, and another study reported no association between pet ownership and depressive symptoms among older adults [25]. Moreover, the relationship between pet ownership and better cognitive functioning in older adults is also contested [26].

Considering that symptoms of anxiety and depression are common among older adults [5] and that preventing cognitive decline is essential for healthy aging [27], it is plausible that pet ownership during this stage of life may contribute to successful aging. However, given the inconsistent findings of previous studies regarding the influence of pets on psychological health and cognition in older adults, further research is warranted. It is also noteworthy that cognitive reserve has been rarely explored in the context of human-animal interaction, underscoring the importance of including this variable in future investigations. Therefore, the aim of this study was to compare symptoms of depression, anxiety, and levels of psychological well-being between older adults who owned pets and those who did not, as well as to examine differences in cognitive functioning and cognitive reserve between these 2 groups.

## Methods

### Design

This is a quantitative cross-sectional observational study.

### Participants

A total of 221 individuals were recruited from the general community in Brazil, primarily from the southern region. Recruitment occurred through social media platforms (Facebook and Instagram). In addition, part of the sample was recruited from existing contact lists of older adults who had previously participated in research studies conducted at the Pontifical Catholic University of Rio Grande do Sul. Posts and messages contained standardized text explaining the study objectives, eligibility criteria, and a contact link for interested participants. Individuals who expressed interest were contacted individually via WhatsApp to confirm eligibility before scheduling the interview. The participants were divided into 2 groups: 1 group consisted of participants who owned pets and the other consisted of individuals who did not own pets.

Eligible participants were community-dwelling older adults aged 59 years or older, who scored more than 14 points on the Modified Telephone Interview for Cognitive Status (TICS-M) [28], were literate, and did not have uncorrected hearing problems that could interfere with completing the instruments. All participants provided electronic informed consent before data collection. A total of 6 individuals who did not complete the entire questionnaire were excluded from the study. The final sample consisted of 215 participants.

### Measures

The Sociodemographic Questionnaire was used to collect information regarding the sociodemographic characteristics of the sample.

The TICS-M was employed to assess the cognitive functions of the participants. The original 41-item version (TICS) was developed to identify cognitive decline through a telephone interview [29]. In its modified version (TICS-M), 2 items were altered to include a measure to assess late memory [30]. This instrument has been translated and validated for the Brazilian population in a sample of elderly stroke survivors, showing a Cronbach α coefficient of 0.93. The Brazilian version of the questionnaire identified 3 domains: working memory, recent and late memory, and orientation [28].

Cognitive Reserve Index Questionnaire (CRIq) was applied to assess the cognitive reserve of the participants. This questionnaire consists of 20 items grouped into 3 subscales (CRIq Education, CRIq WorkingActivity, and CRIq LeisureTime). The total score of the instrument is calculated by adding up the subscores of the 3 subscales. The original version of the CRIq has a Cronbach α coefficient of 0.73 [31], and this questionnaire already has a version translated into Brazilian Portuguese [32], which was used in this study.

Mental Health Continuum-Short Form (MHC-SF) was used to assess positive mental health. It consists of 14 items presented in a Likert scale format, ranging from 1 (never) to 6 (every day). The MHC-SF has 3 subscales that assess subjective or emotional well-being, psychological well-being, and social well-being [33]. In this study, we applied the translated and validated version of the MHC-SF for the Brazilian population, which shows adequate reliability with a Cronbach α of 0.96 [34].

Geriatric Depression Scale (GDS-15) was used to measure the participants’ depression symptoms. The GDS is specifically designed for older adults and addresses depression symptoms experienced in the week preceding the questionnaire application. The short version consists of 15 items, with responses classified as “yes” or “no.” The total score is calculated by adding up the responses selected by the respondents [35]. In this study, we used the translated and validated version for the Brazilian context, which presents adequate reliability coefficients according to the paired Wilcoxon test (*z*=1.60; *P*=.11), Spearman correlation (ρ=0.86; *P*<.001), and weighted kappa (κ=0.64) [36].

Geriatric Anxiety Inventory (GAI) was used to assess symptoms of anxiety. This instrument consists of 20 items in which participants indicate whether they agree or disagree with the statements, considering the week before the questionnaire was applied [37]. In this study, we used the translated and validated version for the Brazilian population, which has a Cronbach α coefficient of 0.90 and significant test-retest reliability (ρ=0.85, *P*<.001) [38].

### Data Collection

Data collection was conducted through individual video calls using the WhatsApp messaging app. During each session, participants’ responses were entered in real time into the Qualtrics platform by the researcher. All data were collected by trained psychology students who received prior instruction and supervision to ensure the standardized administration of the instruments. The data collection period extended from April to September 2023.

### Ethical Considerations

The study was approved by the Research Ethics Committee of the Pontifical Catholic University of Rio Grande do Sul (approval number: 65996522.5.0000.5336) and complied with national and institutional ethical standards for research involving human participants, in accordance with Resolution No. 466/2012 of the Brazilian National Health Council and Resolution No. 016/2000 of the Federal Council of Psychology. All participants received an electronic informed consent form outlining the study’s objectives, potential risks and benefits, anonymity, and voluntary nature of participation. Only those who agreed to the consent form were included in the study. Participants were assured of the confidentiality of their data and their right to withdraw from the study at any time without any consequences. Data were collected and stored anonymously, and no personally identifying information was retained or linked to participant responses. Participants did not receive financial compensation or any other form of remuneration for their participation.

### Data Analysis

The data were analyzed using the JASP software (version 0.18.3.0. JASP Team). Quantitative variables were described using means and standard deviations, while categorical variables were presented in terms of absolute and relative frequency. The normality of the univariate distribution of the variables under investigation was assessed using the Shapiro-Wilk test, adopting a significance level of *P*<.05. Since all variables in this study violated the assumption of normality, nonparametric statistical tests were employed. To compare the groups, the Mann-Whitney *U* test was conducted, followed by a rank-biserial correlation (*r_b_*) to assess the magnitude of the effect size found.

## Results

The final sample of this study consisted of 215 participants, with an average age of 69.13 years (SD 6.89), ranging from 59 to 93 years. Among the participants, 160 (74.4%) were female, 188 (86.2%) resided in the state of Rio Grande do Sul, 113 (51.8%) were married or in a common-law marriage, and 80 (37.2%) had higher education. Regarding pet ownership, 114 (53.0%) were pet owners of at least 1 animal, and 101 (46.9%) did not have pets. Among the participants who reported having pets, the majority (n=88, 77.2%) were female, and the most common marital status was married or in a common-law marriage (n=66, 57.9%). Among those who did not have pets, most (n=72, 71.3%) were female, and 47 (46.5%) reported being married or in a common-law marriage. These and other sociodemographic characteristics are presented in Table 1.

Within the group of pet owners, 18 (8.4%) only had cats, 66 (30.7%) only had dogs, and 3 (1.4%) only owned other species of animals. Additionally, 27 (12.5%) individuals reported owning both dogs and cats; 9 (4.2%) owned dogs and other species; 2 (0.9%) had cats and other species; and 4 (1.9%) were owners of dogs, cats, and other species. The most frequently mentioned other animal species were rabbits, turtles, chickens, and monk parakeets.

Using the Mann-Whitney *U* test, we found significant differences between the groups regarding the total GDS score (*U*=4714, *P*=.02), with a small effect size observed (*r_b_*=−0.18). No significant differences were found in the other variables. The information is provided in Table 2.

**Table 1.** Sociodemographic characteristics of older adults (≥59 y) participating in a cross-sectional study in Brazil.^a^

Variables	Total, n (%)	Pet owners, n (%)	Non–pet owners, n (%)
Gender			
Women	160 (74.4)	88 (77.2)	72 (71.3)
Male	55 (25.2)	26 (22.8)	29 (28.7)
States of the country			
Goiás	1 (0.4)	0 (0)	1 (0.9)
Minas Gerais	6 (2.7)	5 (4.4)	1 (0.9)
Paraná	3 (1.4)	2 (1.7)	1 (0.9)
Rio de Janeiro	2 (0.9)	0 (0)	2 (1.9)
Rio Grande do Sul	188 (86.2)	99 (86.8)	89 (88.1)
Santa Catarina	7 (3.2)	6 (5.2)	1 (0.9)
São Paulo	8 (3.7)	2 (1.7)	6 (5.9)
Marital status			
Married or common-law marriage	113 (51.8)	66 (57.9)	47 (46.5)
Divorced or separated	39 (17.9)	16 (14.0)	23 (22.8)
Single	25 (11.5)	15 (13.1)	10 (9.9)
Widow or widower	38 (17.4)	17 (14.9)	21 (20.8)
Education level			
Incomplete primary education (up to 4 y of study)	8 (3.7)	3 (2.6)	5 (5.0)
Incomplete secondary education (less than 11 y of study)	10 (4.7)	5 (4.4)	5 (5.0)
Complete secondary education (up to 11 y of study)	38 (17.7)	21 (18.4)	17 (16.8)
Incomplete higher education	18 (8.4)	9 (7.9)	9 (8.9)
Complete higher education	80 (37.2)	41 (36.0)	39 (38.6)
Postgraduate (specialization, master’s, or doctorate)	61 (28.4)	35 (30.7)	26 (25.7)
Employment status			
Employed	15 (7.0)	9 (7.9)	6 (5.9)
Self-employed	11 (5.1)	10 (8.8)	1 (1.0)
Retired	146 (67.9)	74 (64.9)	72 (71.3)
Retired but still working	40 (18.6)	20 (17.5)	20 (19.8)
Never worked	3 (1.4)	1 (0.9)	2 (2.0)
Medication use			
Yes	175 (80.3)	89 (78.1)	86 (85.1)
No	40 (18.3)	25 (21.9)	15 (14.8)

a Data were collected through online interviews conducted via WhatsApp video calls between April and September 2023. The table presents the total sample and subgroup distributions for pet owners and nonowners.

**Table 2.** Differences in depression, anxiety, well-being, and cognitive measures between pet owners and nonowners in a cross-sectional study with older adults from Brazil.^a^

Variable	Pet owners (mean)	Non–pet owners (mean)	*U*	*P* value	Effect size	SE effect size	95% CI for rank-biserial correlation	
							Lower	Upper
Orientation TICS-M^b^	6.737	6.752	5651	.75	−0.018	0.079	−0.172	0.136
Recall TICS-M	4.912	4.653	6195	.32	0.076	0.079	−0.079	0.227
Attention TICS-M	4.307	4.495	5462	.48	−0.051	0.079	−0.203	0.103
Memory TICS-M	4.807	4.772	5897	.65	0.024	0.079	−0.130	0.177
Language TICS-M	0.851	0.772	6209	.14	0.079	0.079	−0.076	0.230
Delayed Recall TICS-M	3.123	2.851	6171	.36	0.072	0.079	−0.083	0.223
Total TICS-M	24.737	24.297	5996	.598	0.042	0.079	−0.113	0.194
CRI-Education	126.658	130.059	5265	.28	−0.085	0.079	−0.236	0.069
CRI-WorkingActivity	116.711	116.772	5623	.77	−0.023	0.079	−0.176	0.131
CRI-LeisureTime	110.605	106.030	6272	.26	0.090	0.079	−0.065	0.240
Total CRIq^c^	123.816	123.277	5784	.95	0.005	0.079	−0.149	0.158
EWB^d^ MHC-SF^e^	15.842	15.426	6306	.22	0.095	0.079	−0.059	0.246
SWB^f^ MHC-SF	31.553	31.139	5917	.73	0.028	0.079	−0.144	0.164
PWB^g^ MHC-SF	20.816	20.891	5817	.896	0.010	0.079	−0.127	0.181
Total MHC-SF	68.211	67.455	6113	.43	0.062	0.079	−0.093	0.214
Total GAI^h^	5.325	4.921	6048	.52	0.051	0.079	−0.104	0.203
Total GDS^i^	2.333	3.000	4714	.02	−0.181	0.079	−0.326	−0.028

a Statistical comparisons were conducted using the Mann–Whitney *U* test, with effect sizes reported as rank-biserial correlations (α=.05).

b TICS-M: Modified Telephone Interview for Cognitive Status.

c CRIq: Cognitive Reserve Index Questionnaire.

d EWB: emotional well-being.

e MHC-SF: Mental Health Continuum-Short Form.

f SWB: social well-being.

g PWB: psychological well-being.

h GAI: Geriatric Anxiety Inventory.

i GDS: Geriatric Depression Scale.

## Discussion

### Principal Findings

This study aimed to compare symptoms of depression, anxiety, and levels of psychological and cognitive well-being between older individuals who owned pets and those who did not. The results showed that pet owners presented fewer symptoms of depression compared to non–pet owners, whereas no significant differences were observed for anxiety, cognitive functioning, or cognitive reserve.

The observed association between pet ownership and lower depressive symptoms aligns with previous findings, suggesting that companion animals can mitigate depression symptoms among older individuals who had experienced loss, such as a divorce or the death of a spouse [21]. Furthermore, in another study, older individuals who frequently engaged in activities with their pets reported lower levels of depression symptoms, regardless of factors such as age, gender, and education. However, this effect was not observed when considering only the presence of a pet [39].

Friedmann et al [19] proposed the biopsychosocial model as a framework to explain how companion animals may help minimize depressive symptoms in their owners. According to this perspective, pet ownership can influence health through the interaction of biological, psychological, and social factors. The presence of an animal may enhance the social dimension by providing companionship and emotional support, which can improve the psychological state through social support or other mechanisms, such as reducing depression, stress, and anxiety, and ultimately promote positive health outcomes in older adults [19].

However, not all studies have found the same association. Sharpley et al [40], in a longitudinal study, reported that older individuals with more symptoms of depression were more likely to own a dog. However, in this study, pet ownership was not associated with changes in symptoms of depression over time [40]. Similar results were observed in the study by Mueller et al [41], where pet ownership was associated with a higher likelihood of having experienced symptoms of depression over the course of life. In contrast, no relationship was found between current depression and pet ownership [41].

In another investigation, cat owners exhibited more symptoms of depression in comparison to dog owners or older individuals without pets [42]. These results were corroborated by other studies that also found no evidence of an association between having a pet and symptoms of depression in the older population [19,24,43,44].

One possible explanation for these inconsistencies is that while some older individuals may benefit from living with pets, for others, the responsibility of caring for an animal could be detrimental [24]. This may be due to the stress caused by the commitment or difficulties in meeting the pet’s needs [42]. The responsibility of taking care of a pet might interfere with the individual’s freedom, limiting outings and travel. Financial concerns, such as the costs of food and veterinary care, may also cause worries for some pet owners [45,46].

As for anxiety symptoms, no significant differences were found between the groups. This result also shows contradictory findings across studies. While some research has not observed differences in anxiety symptoms when comparing older individuals with and without pets [19,47], the results from Bolstad et al [48] suggest that having a pet during old age is associated with lower anxiety symptoms. Additionally, the systematic review conducted by Hughes et al [49] concluded that interaction with companion animals can alleviate anxiety symptoms in older individuals. Moreover, a study that evaluated mental health outcomes in older individuals with dogs concluded that the frequency of contact with a dog might be associated with lower anxiety levels [20].

Regarding psychological well-being, the results of this study did not indicate significant differences between the groups. This finding is consistent with previous studies that also found no differences when comparing the psychological well-being of older individuals with and without pets [19,47]. However, the study by Hui Gan et al [46] does not support these findings, highlighting the positive effect of pets on the well-being of older individuals. Furthermore, recent research suggests that owning a dog may be effective in enhancing the psychological well-being of socially isolated older individuals. However, the authors of the study highlight that this effect was not observed among cat owners [50].

As observed by Bennett et al [47], merely owning a pet may not be associated with substantial differences in the well-being of older individuals, a point also noted by studies investigating other psychological measures, such as anxiety and depression symptoms [39,47]. This observation may help explain the inconsistencies in the scientific literature that evaluated human-animal interactions [47]. Recent research conducted with adults supports this hypothesis, indicating that interactions with animals may have a protective effect against negative feelings. These studies show that tactile interactions and activities like playing with, caring for, and training the animal are associated with greater well-being among pet owners [51-53]. Thus, it may be more important to consider the frequency of contact with the pet rather than merely evaluating the presence of a pet [47].

Additionally, comparing these studies is challenging due to their methodological differences, such as the use of different instruments to assess the same constructs and the heterogeneity of their samples. It is also important to consider that mental health is influenced by various factors, and pet ownership is just 1 aspect. Therefore, it is crucial to investigate other elements that also affect mental health [54]. In this study, these sources of variability were minimized by employing validated assessment instruments and standardized procedures for data collection, as well as by including a relatively homogeneous sample in terms of education and cognitive level.

When assessing the cognitive level and cognitive reserve of the participants, no significant differences were found between the groups. An earlier study also found no association between cognitive function and pet ownership [26]. However, other studies reveal that older individuals who own pets exhibit better cognitive function compared to those who do not [22,23,43]. Furthermore, the study by McDonough et al [55], conducted with participants up to 74 years old, indicated that pet ownership was associated with higher levels of cognition and larger brain structures, especially among dog owners. Thus, we can infer from these studies that pet ownership may also be associated with a higher level of cognitive reserve.

The inconsistencies observed in these research results may be due to the different methods used to assess cognition. Additionally, evidence suggests that long-term pet ownership (more than 5 y) is what might provide cognitive benefits for older individuals [56]. Since the time spent with the pet was not included in the analyses of this study, and some studies did not mention whether this variable was evaluated [43,43], this may be an alternative explanation for the discrepancies found between the results.

However, the lack of significant differences between the groups regarding cognitive level and cognitive reserve reinforces the finding that indicates fewer depression symptoms among pet owners. Considering the relationship between depression symptoms and cognitive decline in older individuals [57,58], the absence of differences in cognitive levels and cognitive reserve suggests that variations in depression symptoms between groups cannot be attributed to differences in cognitive function. Instead, it is more likely a direct result of the influence of pet ownership. Thus, pet ownership may act as a protective factor against depression symptoms in the older population.

### Limitations and Future Directions

These findings should be interpreted with caution due to several limitations. First, the cross-sectional design precludes causal inferences. Second, the data were based on self-reported measures, which may be subject to response bias. Additionally, the study did not assess the duration or nature of pet ownership, factors that could influence psychological outcomes. Moreover, the sample was primarily composed of highly educated older adults with preserved cognitive functioning from southern Brazil, which may limit the generalizability of the results to other populations.

Future studies should investigate the activities performed with pets to gain a better understanding of how pet ownership can influence the psychological health of older individuals. Additionally, future studies must explore this association with larger samples and use a longitudinal design to examine the effect of pet ownership on depressive symptoms over time.

### Conclusions

Despite the mentioned limitations, the results of this study are relevant, as they compared groups with similar cognitive characteristics, suggesting that the differences found may be attributed to the presence of pets. Therefore, the present findings indicate that pet ownership may be associated with fewer depressive symptoms in older adults, although no significant effects were observed on cognitive reserve, cognitive functioning, anxiety, or psychological well-being. These findings highlight the potential role of companion animals as a source of emotional support in later life. Moreover, these findings have practical implications, as encouraging community initiatives that foster human-animal interaction, such as volunteer programs in shelters or animal-assisted physical activities, may help mitigate depressive symptoms in older adults while also promoting animal welfare.

## Abbreviations

CRIq: Cognitive Reserve Index Questionnaire
GAI: Geriatric Anxiety Inventory
MHC-SF: Mental Health Continuum-Short Form
TICS-M: Modified Telephone Interview for Cognitive Status
